# PedLER: Pediatric Longitudinal Experience with Residents

**Published:** 2019-03-13

**Authors:** Danielle Weidman, Ori Scott, Natalie Jewitt, Alisha Jamal, Maya Harel-Sterling, Hosanna Au, Angela Punnett

**Affiliations:** 1 Division of Haematology/Oncology, University of Toronto, Ontario, Canada; 2 Department of Paediatrics, University of Toronto, Ontario, Canada

## Background

Mentorship is pivotal to the professional development of medical students.^1^ Residents play an important role in mentoring medical students, impacting their education and career choice.^2^ However, formal resident-student mentorship has not been well explored. Pediatric Longitudinal Experience with Residents (PedLER) is a unique program at the University of Toronto, designed to foster formal mentorship between pediatric residents and first-year medical students.

The program objectives for students cover early clinical exposure, learning about life in residency, exploring pediatrics, and receiving career guidance. For residents, the program provides an opportunity to gain mentorship skills and experience balancing teaching with clinical work.

### Program development and structure

Interested first-year medical students apply and are chosen by lottery. Total program size is based on the number of available resident volunteers. Students and residents participate in separate orientation sessions during which expectations are explained and information sheets are provided. Student-resident pairs are created randomly and then expected to meet at least four half-days over six months (the longitudinal period), which may involve shadowing in a variety of clinical settings, or mentorship meetings. The opportunity exists for longer-term mentorship should the pair choose. At the end of each academic year, students and residents evaluate the program via an online survey containing questions about feasibility, perceived benefit and impact, and suggestions for improvement. Participants do not formally evaluate each other as mentors and mentees. A committee of faculty and resident leaders oversees the program and the university provides administrative support. As per institutional Quality and Risk Management policies, this project is a routine part of educational design and delivery and did not require Quality Improvement approval. Based on preliminary experience and results, Research Ethics Board (REB) approval was obtained in 2017 for ongoing study.

### Program data

From 2012 through 2018, 210 resident-student pairs have participated in PedLER. Quantitative data is presented in Table 1. Students reported positive and non-intimidating relationships with residents. They felt comfortable asking questions and exploring the benefits and challenges of pediatrics without feeling the need to “impress” their mentor. One student commented, “Participating in PedLER was my extracurricular highlight of the year.” Residents enjoyed the opportunity to meet and mentor enthusiastic students, and felt better equipped to teach students and balance mentoring with working in a busy environment. Some residents commented that participation in PedLER made them reflect on their own practice to ensure they set a good example. Negative comments revolved mostly around scheduling difficulties.

**Table 1:** PedLER participation data and quantitative survey results. Surveys were initiated in 2013-2014 (residents only) and became more detailed over time. (N/A = data is not available)

			2012-2013	2013-2014	2014-2015	2015-2016	2016-2017	2017-2018
Number of Students	Eligible (1^st^ year students)		254	254	254	260	258	261
	Applied		74	91	89	104	139	115
	Placed (% of applied)		15 (20%)	32 (35%)	35 (39%)	43 (41%)	40 (29%)	45 (39%)
	Responded to survey		N/A	N/A	25	41	9	23

Number of Residents	Eligible *		62	61	54	76	80	86
	Participated (% of eligible)		15 (24%)	32 (52%)	35 (65%)	43 (57%)	40 (50%)	45 (52%)
	Responded to survey		N/A	23	19	37	11	24

Number of pairs who met †			N/A	23 (100%)	19 (100%)	37 (100%)	11 (100%)	24 (100%)
Number of pairs who met four of more times†			N/A	17 (74%)	13 (68%)	20 (54%)	5 (45%)	10 (42%)

Anticipate continuing a mentorship relationship		Students	N/A	N/A	N/A	26 (63%)	4 (44%)	15 (65%)
		Residents	N/A	13 (57%)	11 (58%)	22 (59%)	5 (45%)	15 (63%)

Students’ interest in pediatrics		Increased	N/A	N/A	N/A	19 (46%)	4 (44%)	11 (48%)
		Decreased				2 (5%)	0	3 (13%)
		Unchanged				20 (49%)	5 (56%)	9 (39%)

* Eligible residents were PGY-2 to PGY-4 from 2012-2013 to 2014-2015. Starting in 2015-2016, PGY-1 residents were included.

† According to resident survey responses.

### Conclusions and future directions

PedLER provides the opportunity for focused resident-student mentorship beyond a single rotation. The program allows residents to benefit from mentoring experience, and medical students to gain early clinical exposure and insight into residency life. Mentoring relationships between students and residents have the potential to continue beyond the formal program. It would have been helpful to initiate surveys at the program’s inception to obtain data over a longer period of time. Based on our preliminary data suggesting the impact PedLER may have on the career choices of medical students, we plan to undertake further and more rigorous research. We also hope to develop recommendations for future longitudinal resident mentorship programs.
